# Noble metal alloy thin films by atomic layer deposition and rapid Joule heating

**DOI:** 10.1038/s41598-022-06595-9

**Published:** 2022-02-15

**Authors:** Yuanyuan Guo, Yiming Zou, Chunyu Cheng, Leyan Wang, Riko I Made, Ronn Goei, Kwan Wee Tan, Shuzhou Li, Alfred Iing Yoong Tok

**Affiliations:** 1 School of Materials Science and Engineering, Nanyang Technological University, Singapore, 639798 Singapore; 2 Institute of Materials Research and Engineering, A*STAR (Agency for Science Technology and Research), Singapore, 138634 Singapore

**Keywords:** Nanoscale materials, Nanoscience and technology, Chemical engineering

## Abstract

Metal alloys are usually fabricated by melting constituent metals together or sintering metal alloy particles made by high energy ball milling (mechanical alloying). All these methods only allow for bulk alloys to be formed. This manuscript details a new method of fabricating Rhodium–Iridium (Rh–Ir) metal alloy films using atomic layer deposition (ALD) and rapid Joule heating induced alloying that gives functional thin film alloys, enabling conformal thin films with high aspect ratios on 3D nanostructured substrate. In this work, ALD was used to deposit Rh thin film on an Al_2_O_3_ substrate, followed by an Ir overlayer on top of the Rh film. The multilayered structure was then alloyed/sintered using rapid Joule heating. We can precisely control the thickness of the resultant alloy films down to the atomic scale. The Rh–Ir alloy thin films were characterized using scanning and transmission electron microscopy (SEM/TEM) and energy dispersive spectroscopy (EDS) to study their microstructural characteristics which showed the morphology difference before and after rapid Joule heating and confirmed the interdiffusion between Rh and Ir during rapid Joule heating. The diffraction peak shift was observed by Grazing-incidence X-ray diffraction (GIXRD) indicating the formation of Rh–Ir thin film alloys after rapid Joule heating. X-ray photoelectron spectroscopy (XPS) was also carried out and implied the formation of Rh–Ir alloy. Molecular dynamics simulation experiments of Rh–Ir alloys using Large-Scale Atomic/Molecular Massively Parallel Simulator (LAMMPS) were performed to elucidate the alloying mechanism during the rapid heating process, corroborating the experimental results.

## Introduction

Alloys such as steels and bronze have traditionally been applied in engineering applications due to their significantly improved physical and chemical properties as compared to their monometallic counterparts. Many of these applications focus on properties such as high oxidation and corrosion resistance^1,2^, superior wear and fatigue resistance^3^, high thermal stability^1,4^ and excellent electrocatalytic ability^5,6^. Recently, modern alloys have found new applications in industries like biomedical, aerospace industries and energy conversion and storage applications^7,8^. Conventional methods used to fabricate metal alloys typically result in bulk alloys; even with methods like mechanical alloying^9,10^ which give nanostructured bulk alloys that have drawbacks like precisely control of chemical composition, thickness and microstructure.

Thin film deposition techniques like physical vapor deposition (PVD)^11,12^, chemical vapor deposition (CVD) and Atomic Layer Deposition (ALD)^13–16^ have been used to prepare alloy thin films. PVD is a deposition process using an alloy source, but it is limited by directionality and conformality constraints. CVD is challenging for high purity, ultra-thin high aspect ratio films, because the process is not self-limiting, nor can it fabricate films on high aspect ratio substrates. ALD allows high purity, highly conformal on high aspect ratio, 3D patterned substrates at low temperature deposition controlled at the atomic scale. These techniques have been widely used in areas like semiconductor manufacturing, fuel cells, solar cells and batteries due to the high surface area, rich reaction sites of the thin film materials compared to bulk materials. As the requirements for these advanced devices become more stringent, the fabrication methods will be required to deposit conformal and uniform thin films with precisely controlled chemical composition, thickness and surface roughness, on substrates with 3D high aspect ratio surface feature^12,17,18^ all the known techniques today are unable to fabricate such thin film alloys.

Typically, metal alloys are post-processed at high temperatures to ensure uniform atomic mixing and formation of functional crystalline phases. Compared to other heating methods for alloy preparation (spark plasma sintering, pressure less sintering, hot press sintering, microwave sintering), electric Joule heating (EJH) has the advantages of ultrafast heating and rapid cooling (on the orders of 10^3^–10^5^ K/s), allowing precise control of homogeneous elemental composition and distribution as well as nonequilibrium transformation of single-phase alloys by inhibiting phase separation^19,20^. allowing precise control of homogeneous elemental composition and distribution as well as nonequilibrium transformation of single-phase alloys by inhibiting phase separation. It has also been reported that atomic interdiffusion distance is shorter in smaller particle sized metals as compared to the bulk at the same temperature^21^. Similarly, metal thin films with nanometer thickness could also exhibit this same behaviour, where the metal atoms would diffuse fast between the constituent layers^21^. In addition, their melting point would be much lower than that of the corresponding bulk metals due to their nanometer thickness^22^. As such, metallic alloys could be fabricated using alternating layers of thin film constituent metals.

**Figure 1:**
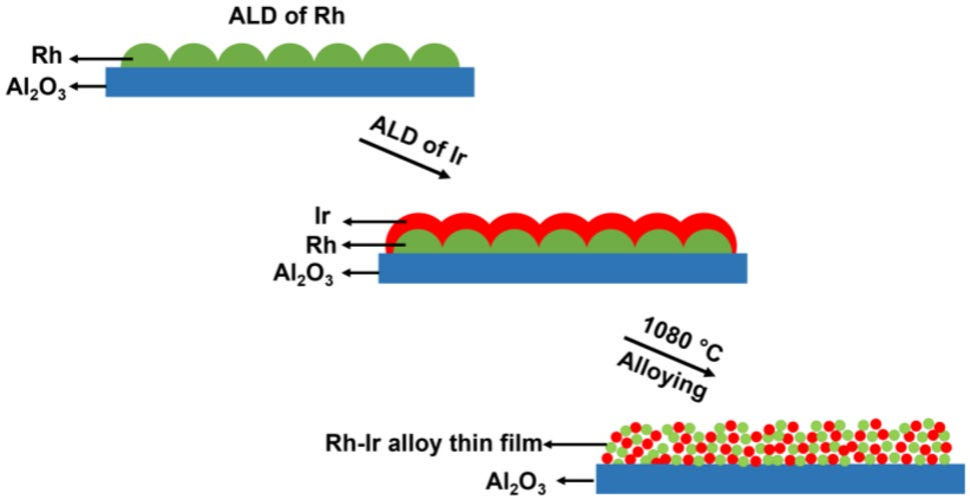
Schematic illustration of the fabrication process of Rh–Ir alloy thin film.

In this work, we demonstrate for the first time a novel strategy consisting of ALD to fabricate alternating layers of nanometer-thin constituent Ir and Rh films on the basis of crystallographic similarity, and subsequent rapid EJH treatment to alloy & sinter these Rh–Ir films into an alloy. After ALD, the multilayered structure was alloyed by EJH at 1080 °C for 5 s to obtain Rh–Ir alloy thin film (Fig. 1). Characterization of the alloy thin film was performed using GIXRD, XPS, TEM and SEM. Molecular Dynamics simulation experiments were employed to elucidate the melting and alloying mechanisms of Rh–Ir alloy thin film.

## Experimental section

### ALD of Rh on sapphire

A custom-built ALD reactor was used to fabricate the thin films on a 1 × 1 cm^2^ sapphire (α-Al_2_O_3_ (0001)) substrate. ALD precursors used for Rh were Rhodium (III) acetylacetonate and ozone as a co-reactant generated in an ozone generator (Nanofrontier, XLK-G20) from high purity oxygen (99.999%). A base pressure was kept at 1.0 mbar by applying 150 sccm nitrogen gas passing through the main chamber. The substrate temperature was kept at 210 °C and the sublimation temperature of Rh precursor was 150 °C. 500 cycles were carried out for Rh ALD. Each cycle was expressed as t1–t2–t4–t1–t2–t4–t3–t4 where t1 is the pulse of carrier gas into the precursor holder (5000 ms), t2 is the pulse of precursor (2000 ms), t3 is the pulse of ozone (300 ms) and t4 is the purging time (2000 ms). A more detailed description of this ALD process conditions and parameters can be found in our previous paper^23^.

### ALD of Ir on Rh

The as prepared Rh on sapphire was loaded in the reaction chamber as substrate and the temperature was kept at 180 °C. ALD precursor used for Ir was Iridium (III) acetylacetonate, and ozone as a co-reactant^24^. The sublimation temperature of Ir precursor was 180 °C. The other operation condition and deposition parameters including cycle number, deposition sequence and pulse/purge times of each reactant were same as used for ALD of Rh^23^.

### Rapid Joule heating of Rh–Ir thin film

The EJH experiments were using a custom-built setup as reported in literature^19–23,25^ under low vacuum conditions (< 1 Torr). The ALD Rh–Ir thin films on sapphire substrates were placed on top of a carbon paper support (Fuel Cell Earth). A tuneable source meter was used to supply potential and current values up to 30 V and 20 A, respectively, to heat the carbon paper. The thin film samples were Joule-heated with a ramp dwell of 3 s and then held at the respective temperatures for 5 s. Finally, the samples were cooled naturally to ambient temperature over 5 min. The peak temperatures of the carbon paper were obtained using a Raytek Raynger thermal infrared sensor.

### Characterization

SEM images were obtained with a field emission gun source (JEOL, 7600F, FESEM) and (ThermoFisher Quatro FE-ESEM). SEM EDX Maps were collected with 20 keV electron energy, to avoid sample damage and carbon deposition, signal collected with short dwell time and multiple frames, i.e.—10 μs and 1000 frames. TEM imaging and EDX mapping were carried out using JEM-2100F (JEOL, Japan), operated at 200 kV, equipped with Oxford EDS Detector. TEM sample were prepared by focused ion beam (FIB) technique, using FEI Nova 600 Nanolabs Dual Beam. The chemical composition and binding information analysis were performed by X-ray photoelectron spectroscopy (Shimadzu Kratos Axis Supra, XPS with 15 kV and 15 mA Al Kα source). The binding energy scale was calibrated automatically with standard samples (silver and gold). The raw XPS data analysis was finished by ESCApe software and the binding energy peak positions were calibrated with C 1 s peak at 284.8 eV. Shirley background and Gaussian–Lorentzian function with 30% Gaussian peak profile were applied for XPS peak fitting. The crystalline structure of the films was examined by grazing incidence X-ray diffraction (Bruker D8 discover XRD System, GI-XRD) using a Cu-Kα radiation with a wavelength of 1.54 Å and a fixed grazing angle (1.0°). The XRR curve was fitted by the software Leptos based on various parameters, including thickness, density, roughness and crystal structure of the thin films.

### Computational details

Rh-core–Ir-shell nanoparticles (Rh–Ir NP) were constructed from a large cubic fcc single crystal to simulate the alloying process of Rh–Ir film. The total number of atoms was 8247 which corresponds to a radius of about 3.1 nm. The Rh percentage is 66.4 at.% according to the experimental results.

Molecular dynamics (MD) simulations were performed using the large-scale atomistic/molecular massively parallel simulator (LAMMPS) open source code^26,27^. The embedded atom method (EAM) potentials^28^ were adopted to depict the interatomic interactions, which describe the lattice parameter, cohesive energy, surface energy, elastic constant, and thermodynamic as well as transitional properties of metals as well as their alloys. The total potential energy of EAM potential is written as^29^:

1 $${\text{E}}_{i} = {\text{F}}_{i} \sum\limits_{i \ne j} {\rho_{ij} } {(}r_{ij} {)}+\sum\limits_{i \ne j} {\varphi_{ij} } {(}r_{ij} {)}$$

where E_i_ and F_i_ are cohesive and embedding energies of atom i, respectively. ρ_ij_(r_ij_) and φ_ij_(r_ij_) are the electron density and pair interaction of j atoms located around the i atom at distance r_ij_.

Firstly, the Rh–Ir NP was first relaxed to a local minimum energy state. After full relaxation, the Rh–Ir NP was heated and constant temperature was employed to allow energy fluctuations. A Nose–Hoover thermostat^30^ was used to control the vibrational temperature for every step. Pressure was not controlled. The Rh–Ir NP was heated including a series of simulations over a temperature range of 27–1927 °C in incremental of 100 °C at a heating rate of 0.5 °C/ps. In addition, a smaller step of 20 °C was employed to study the alloying behaviour more accurately around the melting point. At each temperature, the simulations were carried out for 100 ps.

To further understand the alloying mechanism during the heating process, the Lindemann index was carried out. It rises linearly with the increased temperature in the initial heating stage, and a sharp jump around the melting point is presented. For a system of N atoms, the Lindemann index for the ith atom is written as^31^:

2 $$\delta_{i} = \frac{1}{N - 1}\sum\limits_{j \ne 1} {\frac{{\sqrt {\left\langle {R_{ij}^{2} } \right\rangle - \left\langle {R_{ij} } \right\rangle^{2} } }}{{\left\langle {R_{ij}^{{}} } \right\rangle }}}$$

and the system-averaged Lindemann index is defined as:

3 $$\delta = \frac{1}{N}\sum\limits_{i} {\delta_{i} }$$

where R_ij_ is the distance between the ith and jth atoms. The brackets $\langle {} \rangle$ is the ensemble average at the temperature.

Lindemann index (correlation of the distance between atoms) was used to identify the melting process of Rh–Ir particle. At low temperature (below melting point), Lindemann index increases linearly and slowly with temperature. While around the melting temperature, the index is expected to jump suddenly because of the increased movement of atoms. In this work, the melting process of Rh–Ir particle will be investigated by studying the variation of the Lindemann index with temperature.

## Results and discussion

Figure 1 shows a schematic of the ALD deposition process and EJH for the Rh–Ir alloy thin film fabrication. Rh was deposited as the first layer on Al_2_O_3_ substrate with a thickness of 20 nm as measured by X-ray reflectivity (XRR), followed by the deposition of 10 nm thick Ir overlayer film on the Rh film surface. After that, Rh–Ir alloy thin film was obtained after a EJH process for 5 s at temperature around 1080 °C.

**Figure 2:**
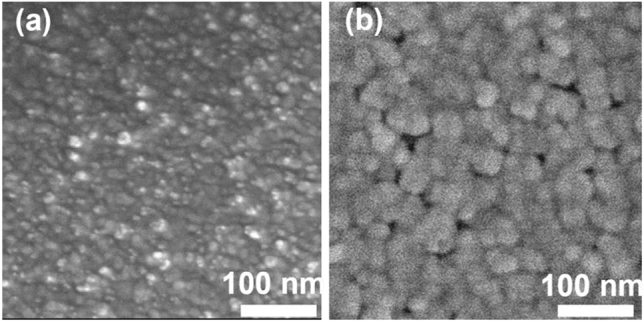
Top-view SEM image of Rh–Ir thin film: (**a**) before EJH; (**b**) after EJH.

SEM was employed to understand the morphology of the as-prepared Rh–Ir thin film before and after EJH. As shown in Fig. 2, the Rh–Ir thin film has spherical morphology before and after EJH. The particle size before EJH is 10.0 ± 3.3 nm based on measurements of at least 100 individual Rh–Ir particles by the SEM image analysis software, whereas the size after EJH induced alloying is larger (31.9 ± 7.7 nm), likely due to the atomic migration, and grain growth^32^. Classic sintering behaviour can be observed, where particles consolidate to form bigger particles (Ostwald ripening) and some voids^33^.

**Figure 3:**
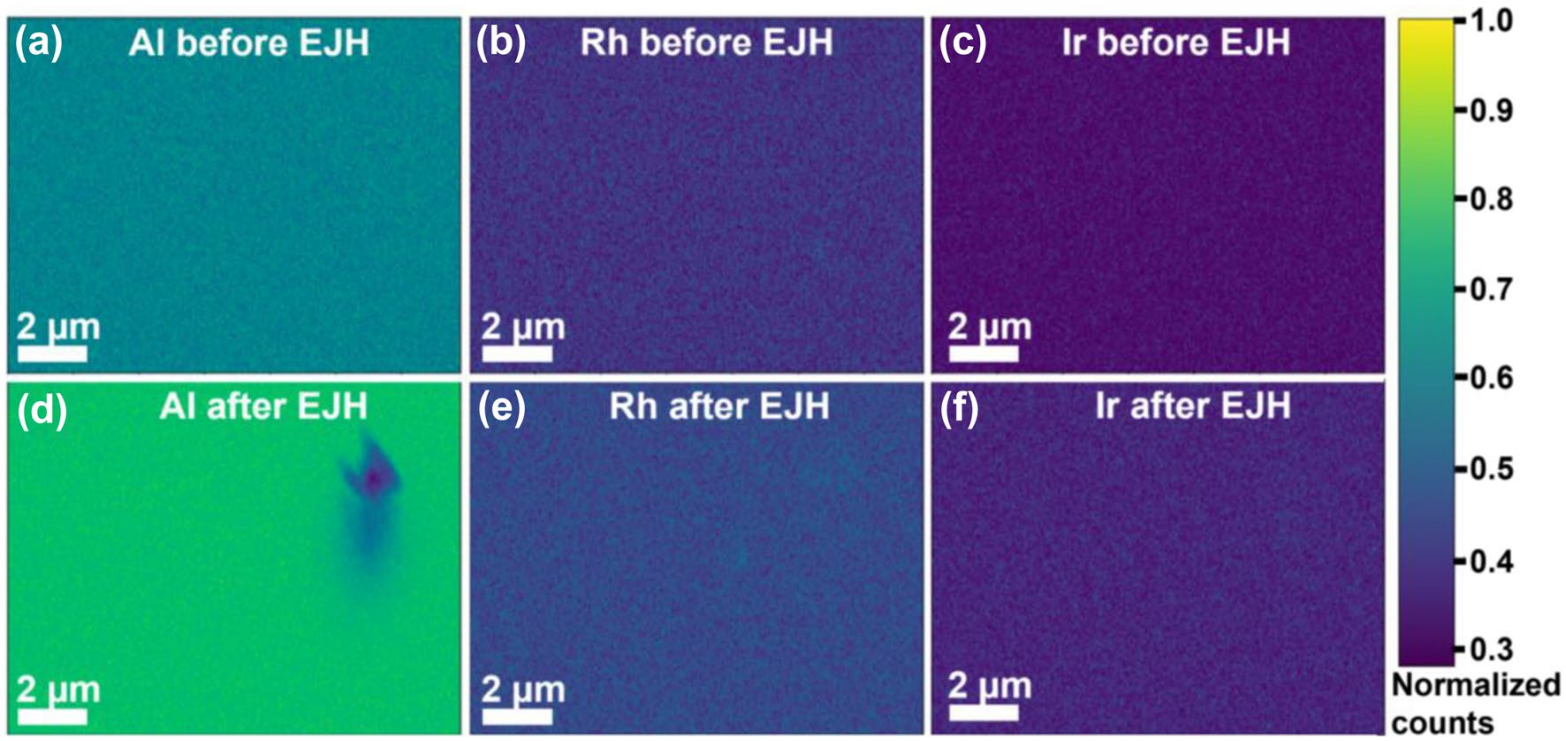
Low magnification EDX elemental maps of Rh–Ir thin film: (**a**–**c**) before EJH; (**d**–**f**) after EJH.

The EDX maps in Fig. 3 show that the constituent elements of the Rh–Ir thin film are homogenously distributed before and after EJH. We did not observe Rh or Ir segregation, which might indicate the formation of an alloy (Fig. 3). On the other hand, X-ray signal from the alloyed sample is significantly higher than the pre-alloyed sample, leading to two possible scenarios. First, the film might become denser after alloying thus improving the electron beam (e-beam) and film interaction. The increase in e-beam-film interaction would then increase the X-ray yield. At the same time, a denser film would also leave localized voids (see Fig. 2b) that allow for X-rays from the substrates to escape easier, reaching the detector and ultimately recorded as brighter. Monte Carlo simulation using CASINO software^34^ showed that 20 keV electron penetrates both Rh and Ir film easily and with minimum interaction. Most of the interaction volume happened at about 1.1um from the film surface, that is probing deeply into the sapphire substrates. The second scenario is Rh–Ir interdiffusion, where Rh diffuses up to the surface and Ir diffuses down towards the substrate. Traditionally, for bulk constituent layers, this scenario would be less likely, as it only possible for a limited range of composition (less than 40 wt.% Rh or more than 90 wt.% Rh), and thermodynamically Rh–Ir system are stable as solid solution at temperature more than 1335 °C^35^. However, as our thin films are only 10–20 nm thickness, we postulate that the atomic interdiffusion distance of Ir and Rh can be similarly reduced as observed in nanoparticles^21^, thereby promoting Rh–Ir interdiffusion to form a more uniform atomic composition in the alloy film and is the most likely cause of the brighter EDX signals observed.

**Figure 4:**
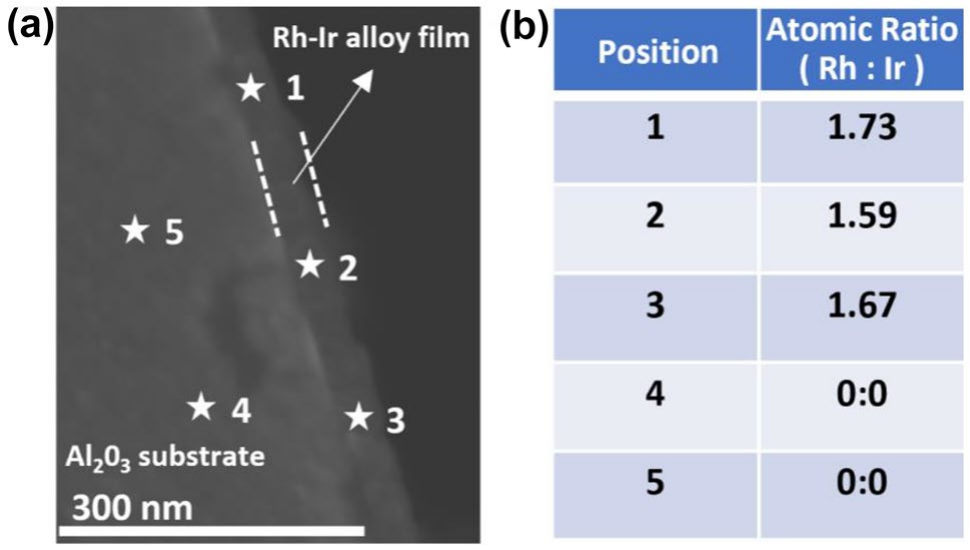
(**a**) Cross-sectional SEM image and (**b**) point elemental analysis of Rh–Ir alloy thin film on Al_2_O_3_ substrate.

Representative elemental compositions at random positions of the Rh–Ir alloy thin film were investigated by point EDX analyses, respectively (Fig. 4). Both Rh and Ir were observed at three random locations (labelled as point 1, 2 and 3) in the metal film with consistent atomic ratio of ~ 1.7 (Fig. 4b), while no Rh or Ir was observed in the Al_2_O_3_ substrate region (points 4 and 5), confirming the diffusion of Rh and Ir.

**Figure 5:**
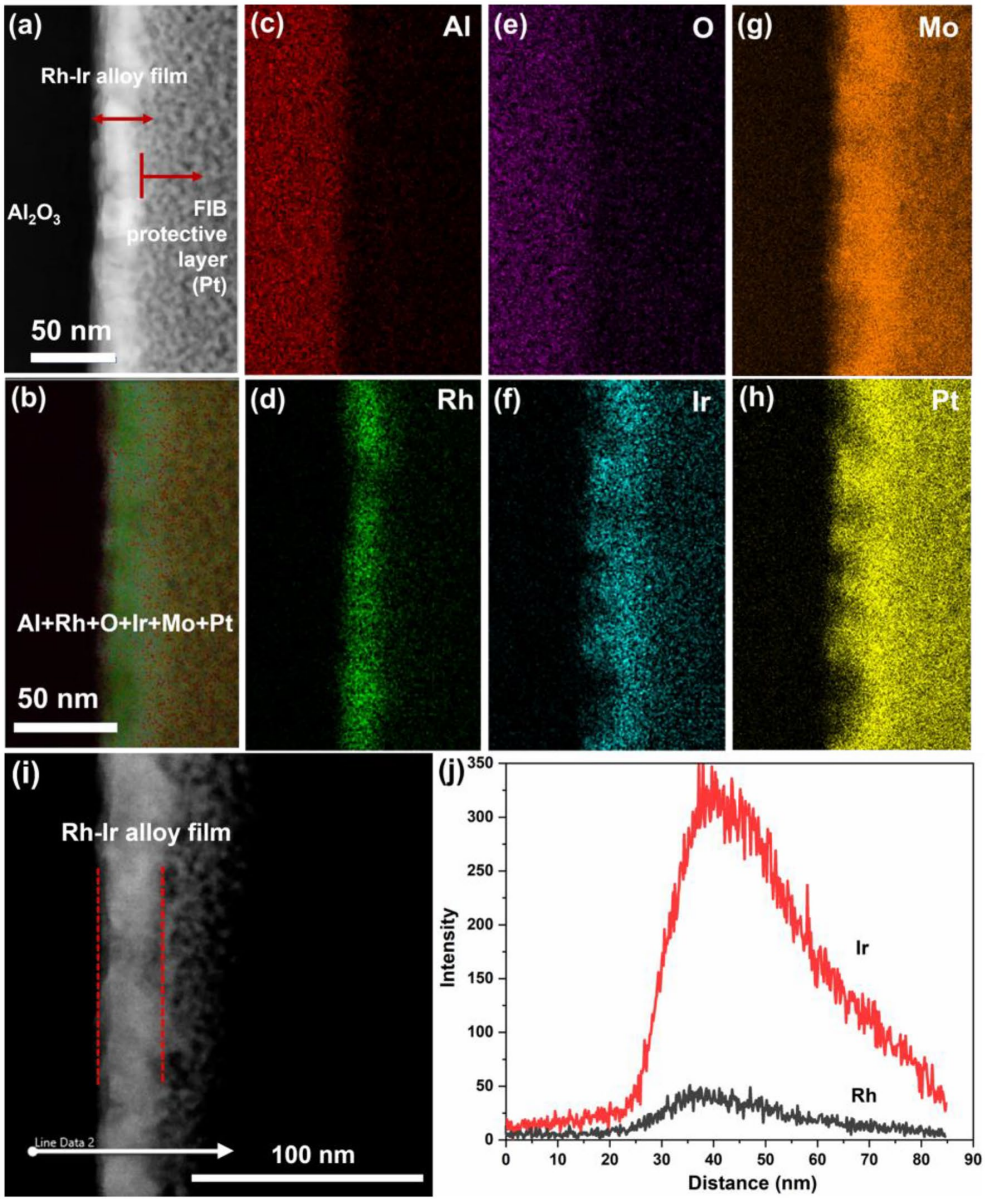
Cross-sectional TEM–EDX elemental mappings of the Rh–Ir alloy thin film. (**a**) Dark field STEM, (**b**) overlapping image, (**c**) Al k⍺1, (**d**) Rh L⍺1, (**e**) O K⍺1, (**f**) Ir M⍺1, (**g**) Mo L⍺1, (**h**) Pt M⍺1. (**i**) TEM image with a line indicating the EDX line scan position. (**j**) EDX line scan with Rh Kα and Ir Kα signals over a distance of 85 nm of the alloy thin film.

The TEM cross-section in Fig. 5a shows a uniformly and conformal Rh–Ir alloy thin film on the Al_2_O_3_ substrate. The total thickness of the Rh–Ir alloy film was measured to be 30 nm, which is in good agreement with that measured by XRR. EDS in mapping mode was used to examine the alloy thin film, indicating that Rh and Ir were relatively well-distributed evenly without obvious evidence of segregation (Fig. 5b–h).

From the STEM micrograph (Fig. 5a), we can observe some brightness variations across the film, which correspond to varying atomic weights. This shows that the alloying parameters have not been fully optimised to obtain full interdiffusion. As such, the EJH processing parameters would have to be studied and optimised to get complete interdiffusion. Nevertheless, it has been shown that alloying did take place to a significant degree.

It is noted to maximize the detection, the X-ray detection range and energy resolution was limited to 10 keV and divided to 1024 channels (0.97 eV/channel). As a result, we are collecting M⍺ lines for Ir instead L-lines (Fig. 5f). Even with these settings, we still experienced Pt M⍺ interference (ΔE = 97 eV) (Fig. 5h). Collecting Ir L⍺ is not preferred as it has much lower intensity, practically far from Rh L⍺ that necessitates to use larger energy range. Consequently, we reduced the energy resolutions.

Furthermore, the formation of Rh–Ir alloy film was also confirmed by TEM line analysis (Fig. 5i,j). The line scan was performed across the thin film and show the overlapping of Rh and Ir along a randomly chosen direction which can be assignable to the formation of alloy structure in the sintered sample^36^. The line intensity is not correlate directly to the element’s concentration, as Ir and Rh has different X-ray emissions yield. We have not calibrated the X-ray signal to for full quantification.

**Figure 6:**
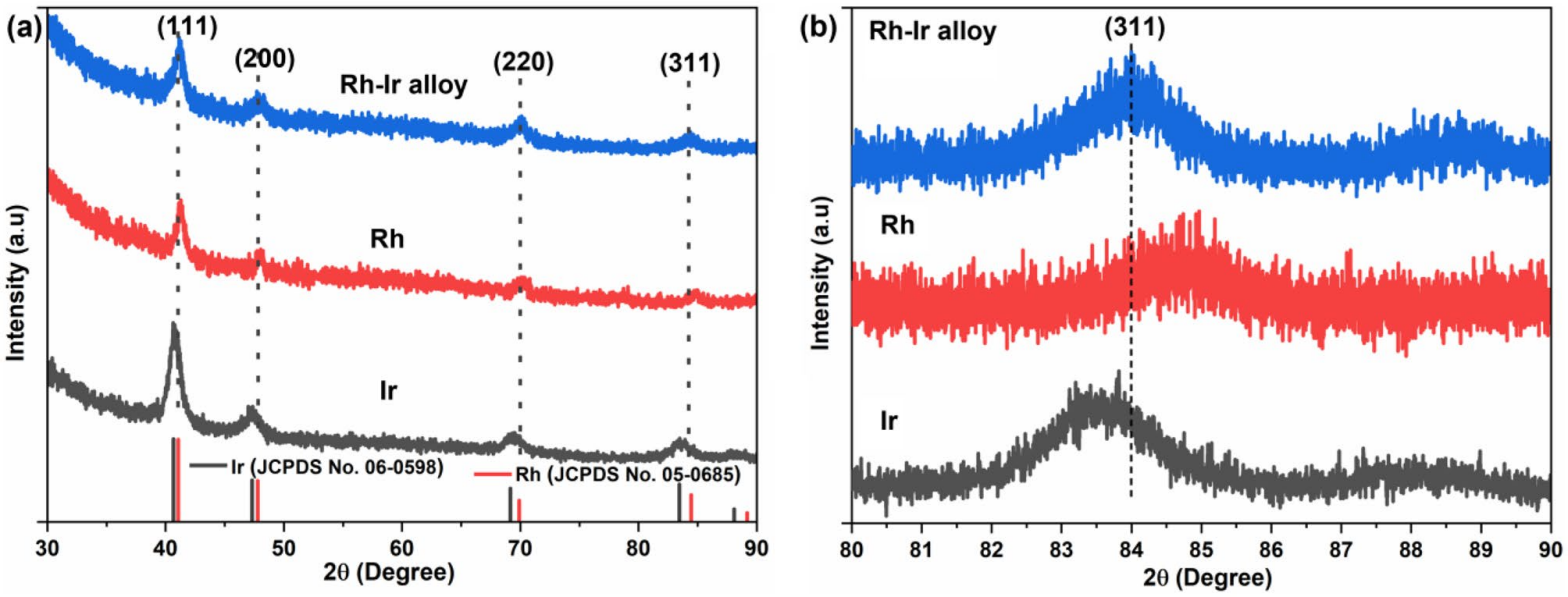
(**a**) XRD patterns of pure Ir film, pure Rh film and Rh–Ir thin film after EJH. (**b**) Slow scan XRD patterns of the (311) peak for pure Ir film, pure Rh film and Rh–Ir thin film after EJH.

Figure 6 shows X-ray diffraction patterns of synthesized pure Rh, Ir and Rh–Ir alloy. Four diffraction peaks are observed, corresponding to the (111), (200), (220) and (311) planes of Rh (JCPDS Card No.: 05-0685) and Ir (JCPDS Card No.: 06-0598) in all diffraction patterns^37^. This indicates that Rh–Ir alloy thin films adopted a face-centered cubic (FCC) structure which is consistent with both pure Rh and Ir thin film.

The peak broadening suggests that the crystallites are very small and finely dispersed. The peak corresponding to the (311) plane for pure Rh is observed at 2θ = 84.5° and that for pure Ir is observed at 2θ = 83.5°. The peak corresponding to (311) plane for Rh–Ir after EJH is observed at 2θ = 84.0° as shown in Fig. 6. The shift in (311) diffraction peak in Rh–Ir alloy thin film confirms the formation of Rh–Ir alloy after EJH^38^. All these peak shifts were also reported by Sampath et al. in the formation of Ir-Rh bimetallic alloy nanoparticles made by hydrothermal method^38^.

**Figure 7:**
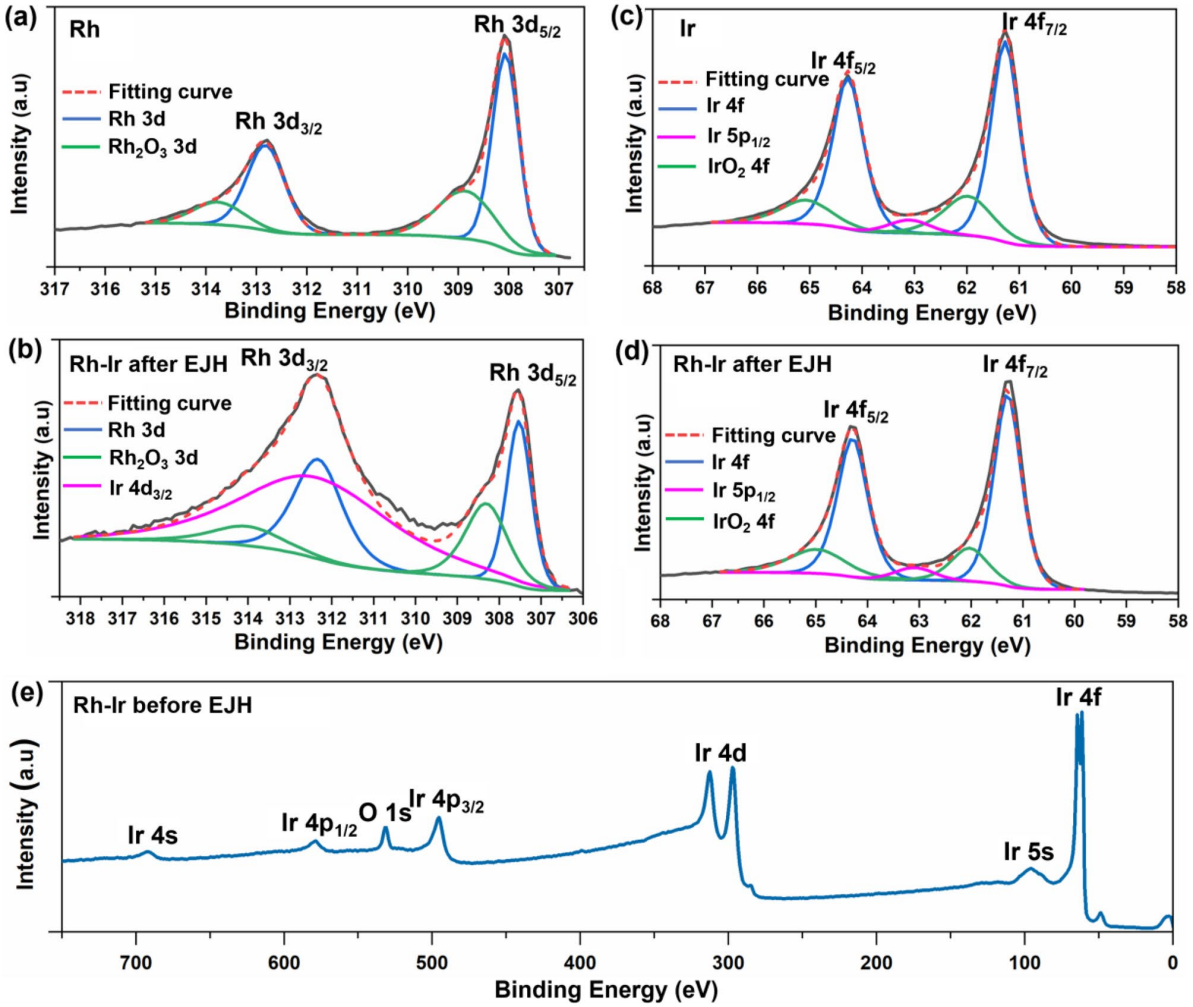
Rh 3d XPS spectra of (**a**) pure Rh and (**b**) Rh–Ir after EJH. Ir 4f XPS spectra of (**c**) pure Ir and (**d**) Rh–Ir after EJH. (**e**) XPS survey spectra of Rh–Ir thin film before EJH.

High resolution XPS spectra Rh 3d and Ir 4f were collected to investigate the local bonding structure and chemical states of the alloy thin film as shown in Fig. 7. The binding energy values of 308.0 eV and 312.8 eV represent Rh 3d_5/2_ and Rh 3d_3/2_ levels of pure Rh in metallic state which is similar with the bonding energy, observed in alloy sample, namely 307.8 eV and 312.6 eV. The spin separations of the Rh 3d for both pure Rh and alloyed Rh–Ir are measured to be 3.0 eV which is characteristic of the metallic Rh. The intensity at 312 eV comes from the overlap of Rh 3d_3/2_ with Ir 4d_3/2_^39,40^. The other values observed at 309 and 314 eV for pure and alloyed samples are consistent with the oxidized species which are attributed to surface oxidation under ambient conditions. The relative ratios reveal that Rh is majority composition in both pure and alloy samples (Fig. 7a,b). Ir spectra for pure Ir and Rh–Ir alloy are shown in Fig. 7c,d. the spectra indicate the presence of metallic Ir with a binding energy of 64.5 and 61.5 eV for pure Ir and at 64.4 eV and 61.4 eV for Rh–Ir alloy. Ir 5p_1/2_ peak is also observed at 63.1 eV which overlaps with the Ir 4f peaks^40^. The relative ratios reveal that metallic Ir is dominant compared to oxides for both Ir and alloy. No significant variation in the intensity of the oxide component was found in Ir before and after alloying. It is worth noting that Rh as the first layer on Al_2_O_3_ was not detected in Rh–Ir thin film before EJH, which was only observed in the sample after EJH, implying the formation of alloy after EJH.

**Figure 8:**
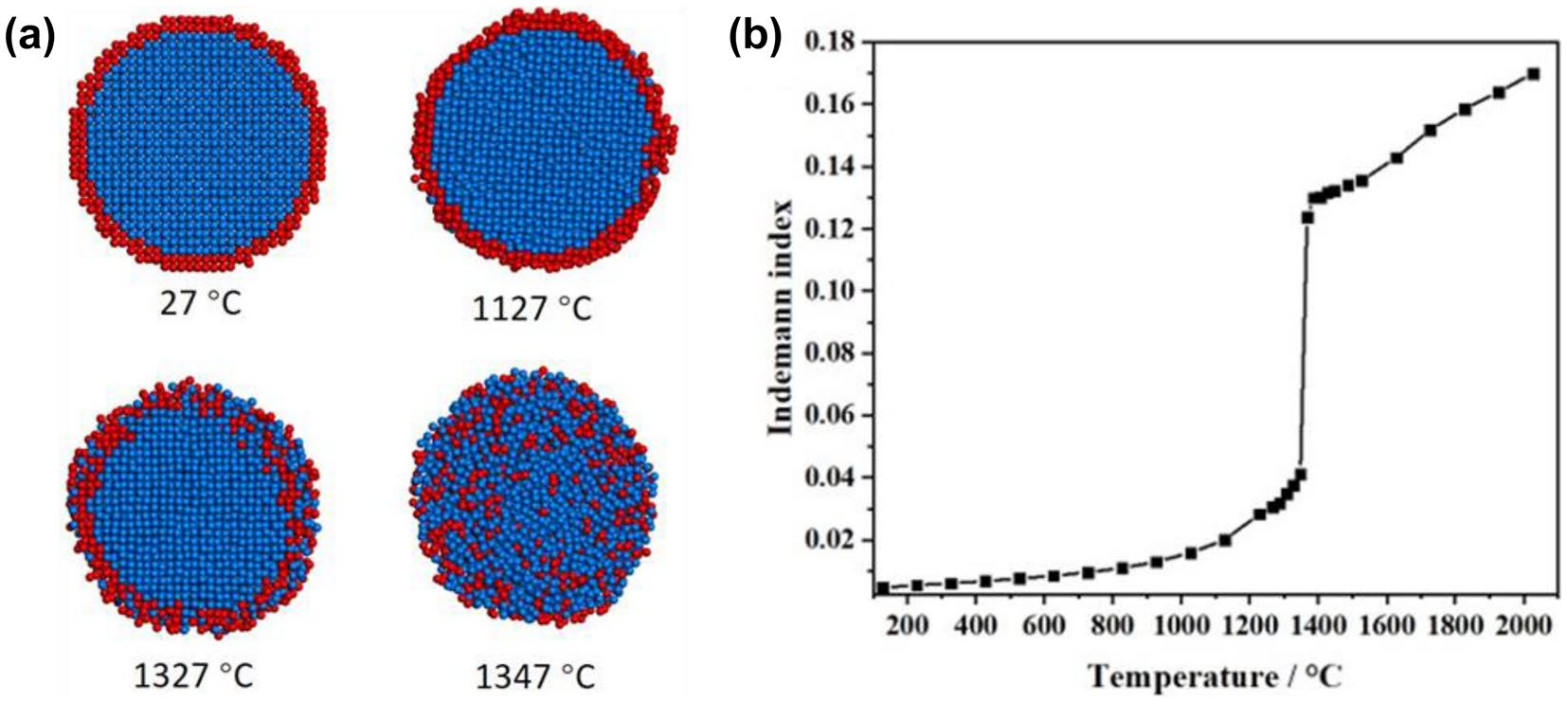
(**a**) Snapshots of Rh–Ir NP taken at four representative temperatures during the heating process and (**b**) the Lindemann indices. Coloring denotes type of atom: red, Ir atom; blue, Rh atom.

From the SEM image (Fig. 2a), it can be seen that the Rh–Ir thin film is constituted by nanoparticles, where Rh is the base layer and Ir is the surface layer. Based on this, MD simulation was carried out using Rh-core @ Ir-shell nanoparticle (Rh–Ir NP) model to further explore the alloying mechanism. Note that melting points of bulk iridium and rhodium of the EAM potential are 2007 ± 15 and 1787 ± 15 °C, which are lower than the experimental values (2466, and 1964 °C, respectively)^41^. Therefore, the melting point of the Rh–Ir NP will be estimated lower than that of the experimental value in scale. Snapshots of Rh–Ir NP taken at four representative temperatures were extracted from MD simulations of the heating process, as shown in Fig. 8. The Lindemann index is illustrated on its right side. At 27 °C, the atoms in the Rh–Ir NP are orderly arranged. When the temperature increases to 1127 °C, only surface pre-melting of Ir shell occurs. Also, Rh core is highly ordered. When the temperature is 1327 °C, Ir melts and diffuses into the Rh core since the thickness of Ir shell is thin. Meanwhile, the internal Rh atoms are still in order. As the temperature reaches 1347 °C, an instantaneous accomplishment of the solid–liquid phase transition of the Rh core occurs. As a result, the alloying of the Rh–Ir NP experiences a quite narrow temperature interval (20 °C, from 1327 to 1347 °C). Subsequently, Ir atoms continue diffusion into the Rh layer and both metal atoms are distributed uniformly, which was in agreement with elemental mapping using TEM (Fig. 5). However, the simulated temperature at which Rh–Ir alloy formed (1347 °C) is ~ 267 °C higher than the experimental one (1080 °C). The possible reasons include: (1) It has been reported that the impurities could lower the melting point of crystals. Noble-metal ALD processes usually produce very pure films with low impurity contents^42^. However, during our experiment, small amount of Rh oxide was observed by XPS. So it is possible the melting point of the Rh–Ir thin film was reduced because of the impurities (metal oxides) in the deposited films^43,44^. (2) As reported in our previous work, some ligand remained in the as deposited film from Rh precursor (Rh(acac)_3_), ALD Rh precursors are likely amorphous with lower melting temperatures than the crystalline counterparts ^23,45,46^.

## Conclusion

In conclusion, ALD technique is capable of fabricate relatively uniform and continuous sub-50-nm thick alloy thin film on 2D substrates compared to other alloy synthesis methods. Our initial work focused on Rh–Ir alloy thin film synthesis involving ALD and EJH alloying techniques. SEM and TEM reveal both Rh and Ir were distributed uniformly in the single-phase solid-solution alloy film with no signs of macroscopic phase segregation on Al_2_O_3_ substrate similar to bulk Rh–Ir alloys prepared by other methods. Optimization of the CTS parameters will be the subject of ongoing works. MD simulations was also applied to investigate the melting mechanism of Rh–Ir thin film. The pre-melting temperature of Ir on the Rh–Ir surface was determined before alloying. As the temperature increase, Ir atoms diffuse into Rh layer, forming a Rh–Ir alloy which is basically in agreement with the experimental characterization results of Rh–Ir alloy thin film. In summary, our proof-of-concept experiments suggests coupling ALD with EJH enables a viable approach to prepare homogenous single-phase solid-solution metal alloy thin films that may find use for various applications in catalysis, microelectronics and other fields.
